# Environment, Autoantibodies, and Autoimmunity

**DOI:** 10.3389/fimmu.2015.00060

**Published:** 2015-02-11

**Authors:** Kenneth Michael Pollard

**Affiliations:** ^1^Department of Molecular and Experimental Medicine, The Scripps Research Institute, La Jolla, CA, USA

**Keywords:** autoantibody, environment, human, animal model

## Introduction

Susceptibility to autoimmune disease is multifactorial and includes genetic predisposition, gender, ethnicity, age, and environment. While no single factor has been identified as preeminent, the role of the environment has garnered increasing interest. This reflects the ubiquitous nature of the environment, which encompasses everything around us including the air we breathe, the water we drink, the food we eat, synthetic and natural chemicals, microorganisms, industrial by-products, and physical factors such as radiation (1). The most convincing evidence for a role of exogenous factors in autoimmunity comes from studies implicating numerous medications in the induction of autoimmune disease, particularly the association of drug-induced systemic lupus erythematosus (SLE) with procainamide and hydralazine (2). Identification of the causal role of medications in the induction of autoimmune disease is due in large part to the fact that medications are taken under medical supervision where drug exposure and possible side effects can be closely monitored. This is not the case with non-therapeutic exposure to environmental factors where contact may include numerous exogenous factors at any particular time. Nonetheless, evidence for the association of (non-therapeutic) environmental exposure with autoimmunity has come from two well documented exposures. In 1981, in Spain, the ingestion of analine adulterated rapeseed oil was linked to a previously unknown disease, subsequently called toxic oil syndrome (TOS), which was characterized by myalgias, peripheral eosinophilia, and pulmonary infiltrates (3). The adulterated oil was sold as “olive oil” by street vendors and subsequently used for cooking. The determination that the adulterated oil was the cause of TOS was based on robust epidemiological evidence. More than 20,000 people were affected and some 2,000 perished. Chronic conditions, including scleroderma and neurologic changes, have been described in the survivors. A clinically similar, though epidemiologically distinct, syndrome was identified in United States in 1989 (3, 4). Eosinophilia myalgia syndrome (EMS) affecting approximately 1,500 individuals was suggested to be due to ingestion of certain lots of l-tryptophan from a single manufacturer. Akin to TOS, EMS is a scleroderma-like syndrome found more frequently in women but unlike TOS was not restricted to a geographical area. The acute phase of the syndrome was characterized by myalgia and eosinophilia, followed by chronic cutaneous lesions, progressive neuropathy, and myopathy. These causative exposures are rare examples in a field hampered by the difficulty of linking putative environmental risk factors with autoimmune disease in humans.

Recently, the National Institute of Environmental Health Sciences (NIEHS) convened an expert panel in a workshop setting to review the role of the environment in the development of autoimmune disease. The meeting addressed specific areas of mechanisms, animal models, epidemiology, diagnostic criteria, and exposure assessment focusing, in particular, on the contribution of chemical, physical, and biological agent exposures; medications were not considered. A series of papers were published summarizing the workshop findings (5–8), and a consensus statement was recently published (9). Together these publications constitute the most recent summary of the state of knowledge on the role of environmental exposures in autoimmune disease. In this opinion piece, I will expand upon some of the findings of the NIEHS workshop and our own studies to examine how environmental exposure can contribute to our understanding of autoimmunity and autoimmune diseases.

## Association between Human Autoimmune Diseases and Environmental Exposures

A significant outcome of the NIEHS workshop was analysis of peer reviewed epidemiology studies of environmental exposures that are associated with autoimmunity in humans from 1980 to 2010 (6). This investigation focused on three broad classes of environmental exposures; chemical, physical, and biological but excluded studies of therapeutic agents, vaccines, and medical devices. Previously established guidelines for environmental exposures and human disease were used to classify exposures as “confident,” “likely,” and “unlikely” to contribute to development of disease based on exposure-disease associations, numbers of studies, established exposure assessment, exposure-response gradient, and evidence of biological plausibility. The most striking of the consensus findings was confidence in the association of silica exposure with several autoimmune diseases including SLE and scleroderma (Table 1). Additional findings included confidence in the linkage between solvents and scleroderma, and smoking and seropositive RA (Table 1). Smoking was considered “likely” to contribute to seronegative RA, SLE, multiple sclerosis, Hashimoto’s thyroiditis, Graves’ disease, and Crohn’s disease, but to be protective against ulcerative colitis. While the NIEHS workshop analysis identified numerous epidemiological studies supporting the contribution of environmental exposure to autoimmune diseases, it also concluded that considerably more research is needed. Greater understanding is required of the types of environmental exposures that lead to autoimmunity, importance of latency period between exposure and presentation of autoimmunity, dose–response relationships, and effects of age, sex, and developmental stage. Significant gaps also exist in our understanding of how genotype (how an individual differs or is specialized within a group of individuals or a species) influences induction of autoimmunity following environmental exposure and how broad is the spectrum of disease phenotypes that develop. Also, important will be greater understanding of how epigenetic mechanisms, including DNA methylation, histone modifications, micro- and long non-coding RNAs, influence gene and disease expression following environmental exposure.

**Table 1:** **Environmental exposures for which there is confidence of an association with human autoimmune disease^a^**.

Category	Agent	Disease	Exposure	Gender
Chemical	Silica	RA	Occupational	Increased risk for males
		SLE	Occupational	Both sexes affected
		Scleroderma (systemic sclerosis)	Occupational	Increased risk for males
		ANCA-associated vasculitis	Occupational	Increased risk for males
	Solvents	Scleroderma (systemic sclerosis)	Occupational	Increased risk for males
	Smoking	Seropositive RA	Smoking history	Increased risk for both sexes
Physical	Sunlight	Multiple sclerosis (exposure protective)	Various	Global none; Canada female, New Zealand female
Biological	Gluten	Gluten-sensitive enteropathy (celiac disease)	Diet	Increased risk for females

*^a^Adapted from Ref. (1, 6)*.

Another limitation in our understanding of environmental-induced autoimmune diseases is the development of criteria that can be used to diagnose and treat autoimmune disease phenotypes resulting from environmental exposure (7). This is likely to be a particularly difficult task and will require consideration of many lines of evidence including clinical, serologic, genetic, epigenetic, and other features. Fortunately, our current understanding of environment-exposure associations provides some help. For example, many autoimmune diseases show a female predominance particularly SLE, scleroderma, and rheumatoid arthritis (RA) where up to 90% of patients may be female (10). However, this is often not observed when studies consider environmental exposures (1). This often reflects the occupational or other gender biased social and cultural activities associated with exposure (Table 1). Thus, silica exposure is associated with greater risk of autoimmune disease in males due to occupational bias of males in dusty trades such as mining (11). Such distinctions allow us to identify relevant cohorts to study in order to more accurately define appropriate diagnostic criteria.

## Environmental Exposure and Mechanisms of Autoimmunity

Little is known about the mechanisms that lead to autoimmunity following environmental exposure (8, 12). This is due in large part to a lack of accepted criteria for diagnosis and/or classification of environmentally associated autoimmunity (7). Mechanistic studies are also hampered by the paucity of animal models that faithfully mimic human exposure, an excellent example being the failure to reproduce TOS in mice. Furthermore, although animal models have provided insight to our understanding of disease mechanisms (e.g., the pristane-induced lupus model), they often recapitulate only some of the facets of human disease (5). In contrast, a wide range of environmental agents have been shown to aggravate disease in autoimmune-prone mice, arguing that genetic susceptibility can be influenced by environmental exposure (5). Such disease exacerbation in a genetically susceptible host supports the importance of gene–environment interactions in autoimmunity.

Limited data from human studies suggest that environmental agents elicit both common and unique features of autoimmunity, and this is supported by animal studies (9). For example, the association of silica exposure with SLE is supported by the presence of disease relevant autoantibodies in humans and mice as well as alterations in T cells subsets. Similarly, the effects of the solvent trichloroethylene (TCE) on cytokine profiles observed in humans have been recapitulated in animal studies. Moreover, both silica and TCE exacerbate systemic autoimmunity in lupus-prone strains. In the case of seropositive RA and smoking, the observed increase of heat shock protein expression and disease associated autoantibodies, such as rheumatoid factor (RF) and anti-HSP70, have been observed in animal studies. The common outcome of autoantibody production clearly suggests that environmental factors influence adaptive immune responses. However, the resulting autoantibody profiles suggest that different exposures lead to responses against different autoantigens. Such exposure-specific responses may be useful diagnostic markers and may provide some insight into disease mechanisms (13).

## Environmental Exposures Can Lead to Autoimmunity but Not Autoimmune Disease

For some exposures evidence of immune activation and/or autoantibodies is not linked to any specific autoimmune disease (6). Asbestos (a silicate) exposure is associated with elevated immunoglobulins, anti-nuclear antibodies (ANA), and RF, but not strong evidence of specific autoimmune disease. Anti-thyroid antibodies have been found following radiation exposure, from the nuclear accident at the Chernobyl Nuclear Power Plant in Ukraine, during infancy or early childhood without evidence of thyroiditis. Mercury exposure from artisanal gold mining is associated with features of immune activation and autoimmunity including proinflammatory cytokines and ANA, but the presence of autoimmune disease has not been established.

Although there is little epidemiological evidence that mercury exposure is associated with specific autoimmune diseases (6, 14), it does produce non-life threatening autoimmunity in mice (5) that is regulated by several genes known to be important in idiopathic systemic autoimmune disease (12). What limits the severity of mercury-induced autoimmunity in humans and animal models is unknown, but a recent study suggests that mercury exposure by itself may not be sufficient for expression of autoimmunity in humans. Mercury is a ubiquitous toxicant in the Cheyenne River Sioux Tribe (CRST) lands in United States due to mercury in watersheds of CRST as a result of gold mining in the Black Hills of South Dakota. The predominant mercury (Hg) exposure in the CRST population is thought to be via fish consumption. The association of ANA and specific autoantibodies in relation to total blood Hg, and Hg exposure through fish eating and smoking has been examined in a cohort of 75 regular fish consumers (15). Gender, age, and fish eating were significant predictors of total Hg and specific autoantibodies; self-reported smoking was not. Although ANA was not significantly associated with Hg alone, the interactions of gender with Hg and proximity to arsenic deposits were statistically significant. Of the 75 participants, 38 were male and 37 female with 5% of males and 24% of females being ANA positive. Among the specific autoantibodies tested, the most noteworthy were those against Sjögren’s-syndrome-related antigen A components 60 and 52 kDa, Sjögren’s-syndrome-related antigen A 52 kDa alone, centromere proteins A and B (CENP-A/B), immunodominant epitopes of the E2 subunits of the pyruvate dehydrogenase complex (PDC-E2), the branched-chain 2-oxo-acid dehydrogenase complex (BCOADC-E2), and the 2-oxo glutarate dehydrogenase complex (OGDC-E2) (M2 EP/MIT3), and the autoantibodies detected in a primary biliary cirrhosis (PBC) panel consisting of MIT3, and synthetic peptides of nuclear pore glycoprotein-210 (gp210) and nuclear antigen Sp100 (sp100). The most common being autoantibodies to M2 EP (MIT3) (15%) and the PBC panel (24%), which may echo the high rates of idiopathic liver cirrhosis in Sioux communities; whether this reflects an environmental and/or ethnic etiology is unknown. To date (February 2015), no autoimmune diseases have been associated with this cohort. The contribution that additional factors, such as gender and ethnicity, play in development of autoimmunity induced by environmental exposure requires more detailed elaboration.

## Conclusion

Strong evidence that environmental exposure can cause autoimmune disease comes from epidemiologic studies showing the association between a diversity of environmental exposures and an assortment of autoimmune diseases. This is further supported by the observation that gender ratios may differ from that of idiopathic autoimmune disease due to the occupational nature of exposure. Progress, however, in identifying and characterizing the presumably large number of environmental factors is hampered by the lack of accepted criteria for diagnosis and/or classification of environmentally associated autoimmunity. Postulated mechanisms suggest that specific types of exposures lead to specific outcomes in both humans and animal models. Environmental exposures can also commonly result in immune activation and autoimmunity without evidence of a defined autoimmune disease. The best example is exposure to mercury, which in humans and animal models, produces autoimmunity and tissue pathology that is mild in severity. Greater understanding of how different environmental exposures result in different disease phenotypes and varying degrees of severity will help identify the mechanisms and checkpoints that control development of autoimmunity and autoimmune disease.

## Conflict of Interest Statement

The author declares that this manuscript and associated research was done in the absence of any commercial or financial relationships that could be construed as a potential conflict of interest.
